# Supplementary material for: Top-funded digital health companies offering lifestyle interventions for dementia prevention: Company overview and evidence analysis
Source: PLoS One. 2025 May 5;20(5):e0323390. doi: 10.1371/journal.pone.0323390 (PMC12052105; doi:10.1371/journal.pone.0323390)
Supplement: Attachment — Submitted filename: Response-to-reviewers.docx [file pone.0323390.s004.docx]

**Response to the Editor and Reviewers**	Zurich, 07.03.2025

Dear Dr. Konwarh,

We are grateful for the invitation to revise our manuscript “Top-funded digital health companies offering lifestyle interventions for dementia prevention: Company overview and evidence analysis” [PONE-D-24-16712]. We appreciate your and the reviewers’ constructive and knowledgeable feedback, which we have incorporated into our revised manuscript by attending to all comments carefully. We also want to thank you for granting us an extension for resubmission, as we were not able to get the whole team together for the original resubmission deadline in October 2024. It is kindly appreciated.

Please find below our detailed answers to the reviewer comments in blue and additions to the manuscript in **bold.** Overall, we sincerely thank the reviewers for their helpful thoughts, comments, advice, and valuable concrete suggestions for improvement. We hope that the revision of our manuscript substantially advances the paper to move it forward in the process.

We are looking forward to hearing from you and will respond to any further questions and comments that you may have.

Sincerely,

<anonymized>

*On behalf of all authors*

==============

# Reviewer #1:

#R1.1 More clarification of the term “invisible second patients” needed.

Thank you for this suggestion. To our knowledge, “invisible second patients” is a common terminology used in dementia research to refer to caregivers who require their own care, due to their caregiving duties which may see them neglect their own health or needs [1,2]. We have now included a definition and corresponding references to this term in the manuscript as follows:

*“Caregivers are often referred to as “invisible second patients”, as they often do not receive adequate support and care for the tasks which they are required to fulfill, negatively impacting their own health needs and well-being [17,18].” p.4, line 112-14.*

#R1.2 No effective treatment for dementia management is available? (Cite the specific literature, if available, for this particular statement. There are so many pharmacological and non-pharmacological approaches to manage dementia)

Thank you for this remark, which also is consistent with feedback #R2.3 and #R2.7. We have now clarified this statement in the manuscript as follows:

*“Since* ***there is no reversible cure*** *for dementia…” p.3, line 81.*

#R1.3 Methodology section is too limited. More clear explanation is needed.

Thanks a lot for your feedback. We agree that the methodology of market analyses is quite limited generally, and no gold standard framework for conducting such research currently exists. However, the authors followed the Safavi et al. (2019) framework, which we found to provide a structured approach to systematically analyzing different digital health companies [3]. This is an approach that has also been followed by other existing market analyses by Keller et al. (2022) and Castro et al. (2024) [4,5].

#R1.4 In Table No.1 the founding year of each company could have been arranged sequentially.

Thank you for this suggestion, we have now arranged each company according to founding year.

#R1.5 From line 261-263 along with level try to provide the category name like – level 1 – good, level 2 – fair and so on.

Thank you for this suggestion, we provided the category names as

*“level 1* ***(good)*** *… level 2* ***(fair)*** *… level 3* ***(poor)****” p.13, line 369-72.*

#R1.6 268 – it is commonly known to everyone that, people with old age suffer from cognitive impairment (dementia). The outcome(s) of the review should be novel.

Thanks a lot for your feedback. While age is a common risk factor of dementia, there is also young-onset dementia which targets individuals <65 years old and is increasingly on the rise. Additionally, not every older adult who suffers from memory loss is diagnosed with dementia-related cognitive impairment, it may also be age-related memory loss. In the highlighted sentence from the reviewer, we simply report findings of the studies conducted by companies, as a standard reporting procedure of review articles and do not make any new claims.

#R1.7 319- The lack of publication may be due to the young age of the companies. Can personal assumption be made in a systemic review or in any kind of scientific review???

Thank you for this important observation. We agree that a systematic review should avoid personal assumptions. Here, our intention was to contextualize the observed scarcity of publications by drawing on documented patterns in the digital health sector, where younger companies often experience a time lag between product development, market entry, and the generation or publication of clinical evidence.

This phenomenon is well-established in the literature. For example, studies show that a large proportion of digital health companies lack clinical robustness in their early years, with many having no clinical trials or regulatory filings during their initial market presence [6].

To address the reviewer’s concern, we have now rephrased this passage in the manuscript to present these factors explicitly as evidence-informed explanations rather than assumptions, and we have added supporting references to underpin this point:

“**In addition to the well-documented challenges of applying traditional evidence generation methods to digital health interventions [41], previous research suggests that younger companies are less likely to have completed and published full clinical trials, as evidence generation tends to lag behind product launch in fast-moving digital health markets [42]. This time lag between founding and the availability of peer-reviewed evidence could partially explain the low number of eligible publications identified in this study. A similar disconnect between scientific positioning and published evidence has also been observed in other areas of digital health [29,42]**.” p.17, line 446-53.

#R1.8 The paper somewhere failed to highlight the digital initiative in managing dementia or reducing the risk of dementia. Moreover, the improvements were just mentioned in a column under the heading “Change in Proxy”.

Thank you for this important observation. We agree that the link between the identified digital initiatives and their intended role in dementia risk reduction was not sufficiently highlighted in the original manuscript. We have now added a more explicit explanation of what the proxies represent in terms of indirect indicators of risk reduction, for example improvements in cognitive performance or memory function. This helps make clearer that while no studies demonstrated a direct reduction in dementia incidence, the observed proxy improvements are consistent with established dementia prevention mechanisms documented in the literature [7–18].

We included the following paragraphs …

… in the introduction to set expectations:

“**Due to the long preclinical phase of dementia and the challenges associated with directly measuring prevention success within typical study durations [11,12], many lifestyle-based prevention trials rely on proxies such as cognitive performance or memory function [13], or lifestyle adherence to assess short-term effectiveness [14], rather than demonstrating direct reductions in dementia incidence [11-14].**” p.3-4, line 87-104.

… and in the discussion:

“**Most of the identified studies did not measure clinical endpoints or dementia incidence directly, but instead reported changes in proxies, such as cognitive performance [45-51] or memory function [52,53]. Such proxies are commonly used in dementia prevention research, particularly in short-term trials where observing disease onset would not be feasible [54]. While these proxy improvements do not constitute direct evidence of reduced dementia incidence, they are aligned with established mechanisms linking cognitive reserve and lifestyle factors to lower dementia risk [55,56].**” p.18, line 473-79.

#R1.9 The advantage of digital management as compared to in-person cognitive training could have been mentioned. Moreover, even the benefits of DHI’s and how it challenged the existing paradigms remain unclear.

Thank you for this constructive suggestion. We now realized that we, indeed, had not highlighted the advantages of DDLS comparison to in-person cognitive training or lifestyle counseling sufficiently yet and have added an additional paragraph in the introduction:

“**Digital dementia-preventive lifestyle services (DDLS) build on the advantages of DHIs and have the potential to reach larger populations, potentially also those in underserved or rural areas, and to provide continuous support independent of the financial, geographical, or time constraints of traditional face-to-face counseling**.” p.4-5, line 126-31.

#R1.10 Research evidences pertaining to this topic are scarce, still the current paper claims to fill the existing literature gap.

Thank you for this observation. We agree that **the available evidence base on DDLS is currently limited**, which **this study also confirms**. To avoid overstatement, we have **carefully reviewed the framing of our contribution and novelty claims** in the manuscript and **toned down any language that could be perceived as overstating the comprehensiveness or finality of our study’s contribution**.

The **specific gap** we aim to fill is not to **provide conclusive evidence** on the effectiveness of DDLS itself, but rather to **map the current global service landscape, assess the state of published clinical evidence, and identify critical gaps in evidence generation practices**. This should position the paper as a **starting point for further research**, rather than a definitive resolution of the evidence gap itself.

This positioning has now been adapted, hopefully more nuancedly, in …

… abstract:

“The study **serves as an initial step toward understanding how DDLS are currently evaluated in today’s market and highlights the need for a more rigorous evaluation of DDLS effectiveness.**” p.2, line 51-53.

… in the introduction:

“Against the background of upcoming demand for **DDLS** and an increasing number of companies in the field of DHIs, this study aims **to provide an initial mapping of top-funded companies and to assess the current state of published clinical evidence linked to their services** by answering the following research questions: …” p.5, line 131-35.

… and in the discussion:

“The aim of this work **was to provide an initial mapping of the globally top-funded DDLS and to analyze the currently available body of published clinical evidence linked to these services**.” p.16, line 397-98.

#R1.11 The sole aim of the paper somewhere got lost. The paper has lost its track from its central objective. Unnecessary details have been mentioned.

Thank you for your remarks. After addressing all reviewer comments, we completed a major revision of the entire manuscript, ensuring only content relevant for our research aims and central objective were addressed. We hope our new significant changes to the manuscript highlight the efforts that have been made to ensure the aim of the paper is always central to our findings and discussion.

#R1.12 Finally, the scope of the review was found to be scant.

Thank you for your comment. While we agree that the clinical evidence of DDLS might be scant, this does align with our review findings that more research is needed within the digital health domain of dementia research. We hope the findings of our review nonetheless place future researchers and digital health companies to better address the clinical and research gaps, and provide more evidence-based solutions for people with dementia and other cognitive impairments.

# Reviewer 2:

#R2.1 Line 133 Data was collected up to January 2023. Consider updating the search to include the last 20 months?

Thank for you this suggestion. Unfortunately, the team no longer has access to the paid (and expensive) financial databases used in this review. We have tried our best to fill this gap via other sources, however due to conflicting evidence, we found it would not be methodologically sound to update this review - which we agree is extremely unfortunate. However, as there is no other existing market analysis of top-funded digital health companies that offer lifestyle interventions for dementia prevention, we believe this is still an important contribution for researchers and emerging digital health companies.

#R2.2 Line 176 The exclusion of non-risk groups (healthy individuals) seems to contradict the focus on prevention. Surely the target prevention population is dementia-free individuals?

Thank you for pointing out this observation. This was indeed misleading, we excluded, for instance, feasibility or usability pilot studies conducted with non-risk populations such as “healthy college students”, and now make this clearer in the methods section accordingly:

“Exclusions were made for studies on non-dementia conditions (e.g., Parkinson's disease), **usability or pre-studies with non-risk populations (e.g., healthy college students)**, …” p.9 line 261-63.

#R2.3 Line 36 “Lack of disease modifying treatments” is no longer accurate. Suggest rewording.

Thank you for pointing out this important point. In line with feedback #R1.2 and #R2.7, we have now changed this wording to:

*“… lack of a* ***reversible cure.****” p.2, line 36.*

#R2.4 Line 52 The conclusion is quite vague and lacks specific direction, suggest rewording to make clear what the added value of this study is.

Thank you for your suggestion. After going through all reviewer comments and refining our manuscript, we have changed the abstract conclusion statement to:

*“****Our study shows that the level of funding received by companies does not reflect the clinical effectiveness of DDLS.*** *The study* ***serves as an initial step toward understanding how DDLS are currently evaluated in today’s market and highlights the need for a more rigorous evaluation of DDLS effectiveness.****” p.2, line 50-3.*

#R2.5 Line 57 Avoid using the term “suffer” to refer to people living with dementia.

Thank you for pointing out this important point and we completely agree. We apologize for this error, it has been changed to:

*“… are currently affected by dementia, …” p.3, line 77.*

#R2.6 Make clear what is meant by dementia. Alzheimer’s disease? Or all-cause dementia syndromes? Those with a clinical diagnosis? Pathology?

Thank you for this valuable comment. We agree that the conceptual definition of dementia for this study was missing. The study sought to capture all relevant digital lifestyle interventions aimed at dementia prevention, regardless of the specific type of dementia targeted. This broad understanding of dementia was also reflected in our search strategy (cf. Table 1 and Table 2), which deliberately included search terms such as “Alzheimer’s disease”, “cognitive decline”, “memory loss”, “brain aging”, and related terms in addition to the term “dementia” itself.

We have now revised both the introduction and methods sections to clarify that we referred to all-cause dementia syndromes. In the introduction we have added the following paragraph:

“**Dementia is an umbrella term covering a range of neurodegenerative syndromes that cause cognitive decline severe enough to interfere with daily life [1]. While it is common for individuals to experience mixed dementia [2], Alzheimer’s Disease (AD) is the most common cause of dementia [3]. Other prevalent forms include Lewy body dementia, vascular dementia, and frontotemporal disorders [1,3]. The wide spectrum of symptoms associated with dementia depend on its form and include, amongst others, memory loss, confusion, speaking difficulties, disorientation in familiar surroundings and hallucinations [4]. Especially in its initial phases, the diagnosis of dementia can pose challenges and become difficult to distinguish from typical age-related changes [5,6].**” p.3, line 67-75.

In the methods section we now specify the search strategy in the text beyond the exact search strings presented in the tables:

“**Since the search strategy aimed to capture companies offering DDLS targeting all-cause dementias, including AD, vascular dementia, Lewy body dementia, and frontotemporal dementia, the search terms used in both Pitchbook and Crunchbase included not only the term "dementia" but also related terms such as "Alzheimer", "cognitive decline", and "memory loss" to account for the broad range of conditions that fall under the dementia umbrella (see S1 and S2 Tables).**” p.6, line 167-72.

#R2.7 Line 74 “Lack of effective treatments” no longer accurate.

Thank you for highlighting this error (also see our response to reviewer comments’ #R2.3 and #R1.2). We have changed the wording to always read

*“Since* ***there is*** ***no reversible cure*** *for dementia, …” p.3, line 81*.

#R2.8 Line 76 make clear what stage of life is being referred to when you talk about lifestyle. The potential impact of lifestyle changes is not constant across the lifespan.

Thank you for this valuable suggestion. We now specify in the introduction that the lifestyle factors referred to in the context of dementia prevention primarily target midlife and older age, which reflects the evidence base showing that modifiable risk factors become particularly relevant from midlife onwards [7,19]. This stage-specific focus has now been added to the corresponding passage to make explicit that the preventive strategies discussed are not uniformly effective across all stages of life:

“Although dementia risk is strongly correlated with age, studies indicate that lifestyle, **particularly from midlife onwards**, significantly influences individuals’ susceptibility to developing the condition later in life [8]. Modifiable risk factors that influence dementia risk include, among others, physical activity, diet, and **smoking** [9,10]. It is estimated that **45%** of all **dementia can** be prevented through the targeted change of these modifiable risk factors **during midlife and older age [11].**” p.3, line 82-7.

#R2.9 Line 78 Cognitive training is not a well established modifiable risk factor for dementia, so it seems a little odd to include this here, particularly when other risk factors with better evidence are not mentioned.

Thank you for pointing out this helpful oversight. We have now changed cognitive training to one of the more well-known 14 modifiable risk factors; “***smoking***”. p.3, line 85.

#R2.10 The phrase ‘directly influence’ is problematic as the evidence is largely not causal and many interacting factors are involved.

Thank you for your comment. We agree that these risks are not causal and have removed “directly” to only say *“Modifiable risk factors that influence dementia risk…”* *p.3, line 84-5.*

#R2.11 Reference 10 is out of date, suggest replacing with an updated figure from more recent research.

We thank the reviewer for pointing this out. We have now changed this reference to a 2024 report of the Lancet standing Commission, which also found that now 45% of dementia can be prevented by targeting modifiable risk factors [7]:

*“It is estimated that* ***45%*** *of all* ***dementia can*** *be prevented…” p.3, line 85-6.*

#R2.12 Line 98 Suggest specifying ‘published’ clinical evidence – as you’ve not reviewed clinical evidence outside of the academic literature.

Thank you for this suggestion. In line 145, we had already clarified “analysis of published clinical studies”, however we have now also changed the sentence in question by the reviewer to:

*“This methodological approach provides us with a current state of* ***peer-reviewed*** *clinical validation…” p.5, line 142-43.*

#R2.13 Line 99 I didn’t follow this. What is being continuously peer reviewed?

Thank you for your question, we realized the content of this sentence was not clear and have now thus rephrased it. The purpose of this was to share a glimpse of how all authors worked together in a team setting for this analysis, as a form of peer-review:

*“****To ensure reliability and credibility, the research team incorporated a peer debriefing method, as common for qualitative research [27]****.” p.5, line 144-45.*

#R2.14 Line 104 Was the Google search systematic? What was the reason to do this?

Thank you for this question. The Google search was included as a supplementary and exploratory validation step to ensure that companies which might not (yet) be captured in financial databases could still be identified if they had a visible web presence. While this web search was not conducted with the same level of systematic precision as the structured searches in Pitchbook and Crunchbase, it applied the same eligibility criteria during the screening and selection process. Ultimately, the two companies identified via Google were later excluded, meaning the final sample of analyzed companies came entirely from Pitchbook, Crunchbase, and expert interviews.

This supplementary web search is already transparently documented in Figure 1 (the flowchart), ensuring full traceability. We have also slightly revised the methods section to better reflect this supplementary role, clarifying that the primary, systematic search relied on financial databases, with Google used only as a supplementary completeness check.

“**To supplement and further validate the searches on Crunchbase and Pitchbook, we conducted an additional exploratory web search using Google, aiming to identify any highly visible companies potentially not (yet) captured in the financial databases. All companies, regardless of their source of identification, were screened and selected using the same predefined eligibility criteria, ensuring consistency in the final sample. The full selection process in the ‘sample characteristics’ section below**.” p.6, line 178-83.

#R2.15 Line 110 Search terms seem very limited. E.g were Alzheimer*, brain health etc not considered?

Thank you for this comment. Please see our response to comment #R2.6, where we clarify that the search strategy deliberately covered a broad spectrum of terms related to all-cause dementia syndromes, including for instance, Alzheimer’s disease, cognitive decline, or brain health.

#R2.16 Line 121 The abstract suggests this study identifies companies globally, however the method section here excluded solutions not written in English. Surely this will limit the ability to accurately capture relevant solutions globally? Suggest removing the global claims given the methodology, and acknowledging this limitation in the discussion section.

Thank you for raising this important point. Pitchbook and Crunchbase are both global financial databases, covering companies across all major regions, including North America, Europe, Asia-Pacific, and beyond. Both databases aim to comprehensively capture startups and established companies receiving external funding, which makes them well-suited for identifying top-funded companies in the global digital health space.

Therefore, in the initial search, we didn‘t restrict the „location“ field/mask provided by Pitchbook/Crunchbase and, in practice, only three companies were excluded for lacking available information in English (cf. Figure 1), suggesting that the actual impact on the final sample is limited. Nonetheless, we have now adjusted the manuscript to temper global claims in the abstract and to explicitly acknowledge this limitation (further) in the discussion section.

“**As the search was limited to companies with available information in English, the sample may underrepresent companies operating primarily in non-English-speaking markets**, **despite Pitchbook and Crunchbase both being global financial databases**. **In total, three companies were excluded due to lacking English-language information. While the impact on the final sample was minor, this language bias should be considered when interpreting the findings** as **globally representative**.” p. 22, line 596-601.

#R2.17 Line 145 Please outline all reasons for exclusion with the number excluded for each reason. A flowchart may be helpful to illustrate how you reached the final analytical sample from the initial pool.

Thank you for this comment. We would like to highlight that Figure 1 (flow chart of the company selection process) and Figure 2 (flowchart of literature selection process) already provide the PRISMA flowcharts outlining the full screening and selection process, including the number of excluded articles and the reasons for exclusion. To ensure this is more visible to readers, we have now added a direct reference to Figure 1 and Figure 2 in the respective methods section for Study 1 and 2 each, explicitly guiding readers to the flowchart for a detailed overview of the exclusion process:

“**The detailed flow of company inclusion and exclusion, including the number of excluded companies per reason, is illustrated in Fig 1**.” p.8, line 230-31.

… and :

“**The detailed flow of article inclusion and exclusion, including the number of excluded articles per reason, is illustrated in Fig 2**.” p.11, line 292-93.

#R2.18 Line 166 The reliance on the company/ solution name being included in the publication is a limitation of this study which should be acknowledged in the limitations section. The company or solution name will not necessarily be included if the study was conducted by an academic partner, or prior to the solution being marketed. Also please state in the methods whether the company/solution name searched across the title/ abtract? Or author list as well?

Thank you for this valuable comment. We fully agree with the reviewer that our approach to identifying relevant publications by searching for company and/or solution names poses a limitation, especially in cases where studies may have been conducted independently and recognize that our methodological approach favors commercially visible solutions and may underrepresent academic, early-stage, or non-commercial initiatives.

We therefore now explicitly acknowledge this issue in the limitations section of the manuscript and provide additional transparency regarding the search coverage of the chosen databases, Google Scholar and PubMed:

“**Firstly, while Google Scholar search results may include the full text of open access publications, PubMed searches only covered structured fields such as titles, abstracts, author lists, and (for recent publications) author affiliations. As a result, the identification of relevant publications primarily depended on the explicit mention of company or solution names in these searchable fields. This approach may have missed studies conducted by independent academic partners or published prior to product commercialization, where company or product names might be less likely to be referenced. However, for the top-funded companies targeted in this study, we consider the likelihood of explicit product or company name inclusion to be relatively high, as digital health companies typically have a strong commercial interest in showcasing clinical evidence to support regulatory approval, payer negotiations, or market positioning—particularly in regulated health markets [60,61].**” p.22-23, line 606-23.

#R2.19 However, As company websites were reviewed, did you consider contacting the companies to ask for signposting to their clinical evidence?

Thank you for this suggestion. Indeed, as part of a related research effort, **interviews with company representatives were aimed for and conducted** during a related master's thesis project. However, these interviews were designed to explore **business models and strategic positioning**, not to systematically collect clinical evidence related to the two research questions presented in this study.

Eventually, our study aimed to systematically assess **publicly available clinical evidence** rather than relying on company-provided information and we are convinced that relying on publicly available information in databases, scientific publications, and company websites for our research questions ensured a **systematic and unbiased assessment of the available clinical evidence**.

Additionally, the limited response rate (only two out of nine completed interviews) suggests that direct company outreach **may not have yielded a comprehensive or representative dataset for this study**.

#R2.20 Line 171 Again, dementia seems rather limited as a search term on it’s own.

Thank you for this insightful comment. The inclusion of "Dementia" as a search term was a deliberate choice to align with our research objective, and our study’s narrow focus on dementia-preventive lifestyle interventions, rather than general cognitive decline, cognitive impairment, or brain health more broadly. However, we now acknowledge that this approach may have excluded studies on interventions addressing broader cognitive health aspects that indirectly contribute to dementia prevention but do not explicitly mention dementia in the title or abstract. To clarify this point, we have revised the methods section to emphasize that this filter was applied selectively and only when broader searches produced an unmanageable number of results unrelated to dementia prevention.

“**Given that our study specifically examines dementia-preventive lifestyle interventions, the term "Dementia" was used to refine search results and maintain alignment with our research focus. This filter was applied selectively and only in cases where broader searches produced results unrelated to dementia prevention**.” p. 9, line 253-56.

Additionally, we have explicitly recognized this as a limitation in the revised manuscript:

“**Secondly, in some cases, search results were narrowed using the term "Dementia" to focus on DDLS in alignment with our research objective. While necessary for refining search relevance, this approach may have excluded studies examining broader cognitive health interventions that could have indirect relevance to dementia prevention but do not explicitly mention dementia in their title, abstract, or keywords**.” p.23, line 624-28.

#R2.21 Line 174 – Why refer to access to care here? How is this related to prevention?

Thank you for this question. The inclusion of access to care as a relevant outcome measure follows the market analysis framework established by Safavi et al. (2019) [3], which evaluates digital health interventions based on their impact on clinical outcomes, costs, and access to care. Given that our study adopts this framework to assess the role of digital dementia-preventive lifestyle services (DDLS), we maintained access to care as a relevant criterion for evaluating their broader impact.

To clarify this, we have expanded a brief explanation in the methods section, explicitly referencing our alignment with Safavi et al. (2019):

“Identified studies were sought to be relevant if they were peer-reviewed publications that examine the potential effects of the identified solutions on clinical outcome, cost, or access to care in dementia care or dementia prevention, **aligning with the broader impact categories outlined in the market analysis framework by Safavi et al. (2019) [26].**” p.9, line 260-61.

We now further clearly report the results with regards to the access of care outcome as one potential impact form:

**“Of the nine studies assessed for USPSTF level of evidence (clinical effectiveness publications only), all measured impact in terms of patient outcomes, and none did so in terms of cost or access to care**.” p.13, line 367-69.

… and discuss this finding in more detail in the discussion section:

“**Notably, none of the identified studies explicitly measured the impact of DDLS on cost or access to care, despite these being key evaluation criteria in previous digital health market analyses [26]. While clinical effectiveness remains central to prevention, improving access to such interventions is also a critical enabler of their impact. This absence reflects a broader gap in research on how digital solutions enhance accessibility to dementia-preventive interventions, particularly for at-risk populations who face barriers to traditional in-person care, such as geographic limitations, socioeconomic constraints, or healthcare provider shortages.**” p.19, line 507-13.

#R2.22 Line 221 Geographical analysis – I’m not sure what conclusions can be drawn from these results given the language restriction used in the methodology, as mentioned earlier.

Thank you for this suggestion. We already discuss the limitations stemming from our language restrictions in the limitations section here.

“One limitation of the global market analysis is its focus on English-language solutions, excluding companies targeting non-English markets, potentially overlooking well-funded entities.” p. 20, line 420-422 (original submission)

The wording to this limitation has been further amended in line with comment #R2.16 above.

We have now further preempted this limitation and thus implication on findings in the result sections as follows:

**“Our** analysis revealed a significant European presence among the top-funded companies, alongside notable representations from North America and Asia, **however we also consider our language exclusion criteria as a potential limitation – which we discuss further in this paper**.” p.12, line 315-17.

#R2.23 Line 230 This whole paragraph seems to stray away from scientific results from the data collected. Suggest reducing rhetoric not related to the data, and saving interpretation for the discussion section.

Thank you for this remark. We agree that the paragraph was suggestive and have removed it from the results section, where a few elements of this have been moved to the discussion section.

# List of References (used for response to reviewers)

1.	Brodaty H, Donkin M. Family caregivers of people with dementia. Dialogues Clin Neurosci. 2009;11: 217–228. doi:10.31887/DCNS.2009.11.2/HBRODATY

2.	Duplantier SC, Williamson FA. Barriers and Facilitators of Health and Well-Being in Informal Caregivers of Dementia Patients: A Qualitative Study. International Journal of Environmental Research and Public Health 2023, Vol 20, Page 4328. 2023;20: 4328. doi:10.3390/IJERPH20054328

3.	Safavi K, Mathews SC, Bates DW, Dorsey ER, Cohen AB. Top-Funded Digital Health Companies And Their Impact On High-Burden, High-Cost Conditions. Health Aff. 2019;38: 115–123. doi:10.1377/hlthaff.2018.05081

4.	Keller R, Hartmann S, Teepe GW, Lohse KM, Alattas A, Car LT, et al. Digital Behavior Change Interventions for the Prevention and Management of Type 2 Diabetes: Systematic Market Analysis. J Med Internet Res. 2022;24: e33348. doi:10.2196/33348

5.	Castro O, Salamanca-Sanabria A, Alattas A, Teepe GW, Leidenberger K, Fleisch E, et al. Top-funded companies offering digital health interventions for the prevention and treatment of depression: a systematic market analysis. Archives of Public Health. 2024;82: 1–16. doi:10.1186/S13690-024-01424-Z

6.	Day S, Shah V, Kaganoff S, Powelson S, Mathews SC. Assessing the Clinical Robustness of Digital Health Startups: Cross-sectional Observational Analysis. J Med Internet Res. 2022;24: e37677. doi:10.2196/37677

7.	Livingston G, Huntley J, Liu KY, Costafreda SG, Selbæk G, Alladi S, et al. Dementia prevention, intervention, and care: 2024 report of the Lancet standing Commission. The Lancet. 2024;404: 572–628. doi:10.1016/S0140-6736(24)01296-0

8.	Sperling RA, Aisen PS, Beckett LA, Bennett DA, Craft S, Fagan AM, et al. Toward defining the preclinical stages of Alzheimer’s disease: Recommendations from the National Institute on Aging-Alzheimer’s Association workgroups on diagnostic guidelines for Alzheimer’s disease. Alzheimer’s & Dementia. 2011;7: 280–292. doi:10.1016/J.JALZ.2011.03.003

9.	Ngandu T, Lehtisalo J, Solomon A, Levälahti E, Ahtiluoto S, Antikainen R, et al. A 2 year multidomain intervention of diet, exercise, cognitive training, and vascular risk monitoring versus control to prevent cognitive decline in at-risk elderly people (FINGER): a randomised controlled trial. The Lancet. 2015;385: 2255–2263. doi:10.1016/S0140-6736(15)60461-5

10.	Kivipelto M, Mangialasche F, Ngandu T. Lifestyle interventions to prevent cognitive impairment, dementia and Alzheimer disease. Nature Reviews Neurology 2018 14:11. 2018;14: 653–666. doi:10.1038/s41582-018-0070-3

11.	Çinar N, Sahiner TAH. Effects of the online computerized cognitive training program BEYNEX on the cognitive tests of individuals with subjective cognitive impairment and Alzheimer’s disease on rivastigmine therapy. Turk J Med Sci. 2020;50: 231–238. doi:10.3906/sag-1905-244

12.	Sahiner TAH, Cınar N. TD-P-002: EFFECTS OF ONLINE COMPUTERIZED COGNITIVE TRAINING PROGRAM BEYNEX ON THE COGNITIVE TESTS OF INDIVIDUALS WITH SUBJECTIVE COGNITIVE IMPAIRMENT (SCI). Alzheimer’s & Dementia. 2018;14: P186–P186. doi:10.1016/J.JALZ.2018.06.2018

13.	Bahar-Fuchs A, Barendse MEA, Bloom R, Ravona-Springer R, Heymann A, Dabush H, et al. Computerized Cognitive Training for Older Adults at Higher Dementia Risk due to Diabetes: Findings From a Randomized Controlled Trial. The Journals of Gerontology: Series A. 2020;75: 747–754. doi:10.1093/GERONA/GLZ073

14.	Bahar-Fuchs A, Webb S, Bartsch L, Clare L, Rebok G, Cherbuin N, et al. Tailored and Adaptive Computerized Cognitive Training in Older Adults at Risk for Dementia: A Randomized Controlled Trial. Journal of Alzheimer’s Disease. 2017;60: 889–911. doi:10.3233/JAD-170404

15.	Embon-Magal S, Krasovsky T, Doron I, Asraf K, Haimov I, Gil E, et al. The effect of co-dependent (thinking in motion [TIM]) versus single-modality (CogniFit) interventions on cognition and gait among community-dwelling older adults with cognitive impairment: a randomized controlled study. BMC Geriatr. 2022;22: 720. doi:10.1186/S12877-022-03403-X

16.	Gigler KL, Blomeke K, Shatil E, Weintraub S, Reber PJ. Preliminary evidence for the feasibility of athome online cognitive training with older adults. Gerontechnology. 2013;12: 26–35. doi:10.4017/GT.2013.12.1.007.00

17.	Shatil E. Does combined cognitive training and physical activity training enhance cognitive abilities more than either alone? A four-condition randomized controlled trial among healthy older adults. Front Aging Neurosci. 2013;5: 41237. doi:10.3389/FNAGI.2013.00008

18.	Edgar DL, Bargmann PL. Clinical Study of the Effectiveness of Constant Therapy in the Treatment of Clients With Dementia: Implications for Telepractice. Perspect ASHA Spec Interest Groups. 2021;6: 691–703. doi:10.1044/2021_PERSP-20-00123

19.	Baumgart M, Snyder HM, Carrillo MC, Fazio S, Kim H, Johns H. Summary of the evidence on modifiable risk factors for cognitive decline and dementia: A population‐based perspective. Alzheimer’s & Dementia. 2015;11: 718–726. doi:10.1016/j.jalz.2015.05.016
